# Thrombus of the Aorta and SARS-CoV-2 Infection: Cause or Trigger?

**DOI:** 10.3389/fcvm.2021.700292

**Published:** 2021-09-06

**Authors:** Guillaume Goudot, Mourad Amrane, Rida El Ayoubi, Alain Bel, Nicolas Gendron, Lina Khider, Andréanne Durivage, David M. Smadja, Emmanuel Messas, Paul Achouh, Tristan Mirault

**Affiliations:** ^1^Vascular Medicine Department, Georges Pompidou European Hospital, Institut National de la Sante et de la Recherche Medicale (INSERM) U970 Paris Research Cardiovascular Center (PARCC), Assistance Publique Hopitaux de Paris (APHP), Université de Paris, Paris, France; ^2^Cardiac Surgery Department, Georges Pompidou European Hospital, Assistance Publique Hopitaux de Paris (APHP), Université de Paris, Paris, France; ^3^Pathology Department, Georges Pompidou European Hospital, Assistance Publique Hopitaux de Paris (APHP), Université de Paris, Paris, France; ^4^Université de Paris, Innovative Therapies in Hemostasis, Institut National de la Sante et de la Recherche Medicale (INSERM), Paris, France; ^5^Hematology Department and Biosurgical Research Lab (Carpentier Foundation), Assistance Publique Hôpitaux de Paris-Centre-Université de Paris (APHP-CUP), Paris, France

**Keywords:** COVID-19, aortic arch, arterial thrombus, plaque erosion, endothelial dysfunction

## Abstract

**Objective:** Coronavirus disease 19 is a well-established cause of rare arterial thrombosis. Nevertheless, the exact mechanism of arterial thrombosis remains to be elucidated. We herein report the case of a large floating thrombus of the aortic arch, its surgical management and histological analysis.

**Case:** A 65-year-old patient presented to the emergency department with a suspected stroke. He was non-smoker, but presented cardiovascular risk factors, namely hypertension, type 2 diabetes and hyperlipidaemia. A computed tomography of the aorta revealed a large floating thrombus of the aortic arch, at the base of the brachiocephalic trunk, suspected to be the etiology of stroke. Therapeutic anticoagulation was immediately started. The decision was made to perform an open aortic replacement surgery because of the symptomatic thromboembolic event with recent cerebral infarction and the potential harmfulness of the thrombus due to its size. A mobile thrombus was observed at the base of the brachiocephalic trunk by echocardiography. It was attached to a small area of the upper aortic wall and had an irregular surface. Histology revealed a platelet-rich thrombus lying on an aortic atherosclerotic plaque without pronounced inflammation. No plaque ulceration was present but endothelial cell desquamation was observed consistent with plaque erosion.

**Conclusion:** In our case, there was a thrombus lying on an atherosclerotic plaque with intact thick fibrous cap, but associated with a plaque erosion mechanism. The thrombus formation appeared more likely to relate to a very localized endothelial injury.

## Introduction

The severe acute respiratory syndrome coronavirus 2 (SARS-CoV-2) is responsible for the worldwide pandemic coronavirus disease 19 (COVID-19), a systemic disorder presenting typically with fever and respiratory symptoms. However, vascular manifestations, in the form of thrombosis, were rapidly reported. The first Chinese series found significant biological changes in coagulation biomarkers, associated with endothelial damage (1). Furthermore, an increased incidence of deep venous thrombosis and pulmonary embolism associated with COVID-19 has been established (2, 3). Arterial thrombosis has also been reported but much more rarely (4), affecting mainly the descending thoracic and abdominal aorta. Few cases of aortic arch thrombus have been reported but without histopathology specimen (5–9). We herein report the case of a large floating thrombus of the aortic arch, its surgical management and histological analysis.

## Case Description

A 65-year-old patient presented to the emergency department with a suspected stroke. He was a non-smoker, but presented cardiovascular risk factors, namely hypertension, type 2 diabetes and hyperlipidaemia. His usual prescription included valsartan 160 mg, amlodipine 10 mg, spironolactone 25 mg, atenolol 50 mg, chlortalidone 12.5 mg, gliclazide 60 mg, metformin 2,000 mg, vildagliptin 100 mg, and atorvastatin 10 mg per day. Due to his resistant hypertension, the patient had previously participated to a clinical trial testing a carotid baroreceptor stimulation device (Barostim neo®, CVRx) that he was still carrying.

**Figure 1:**
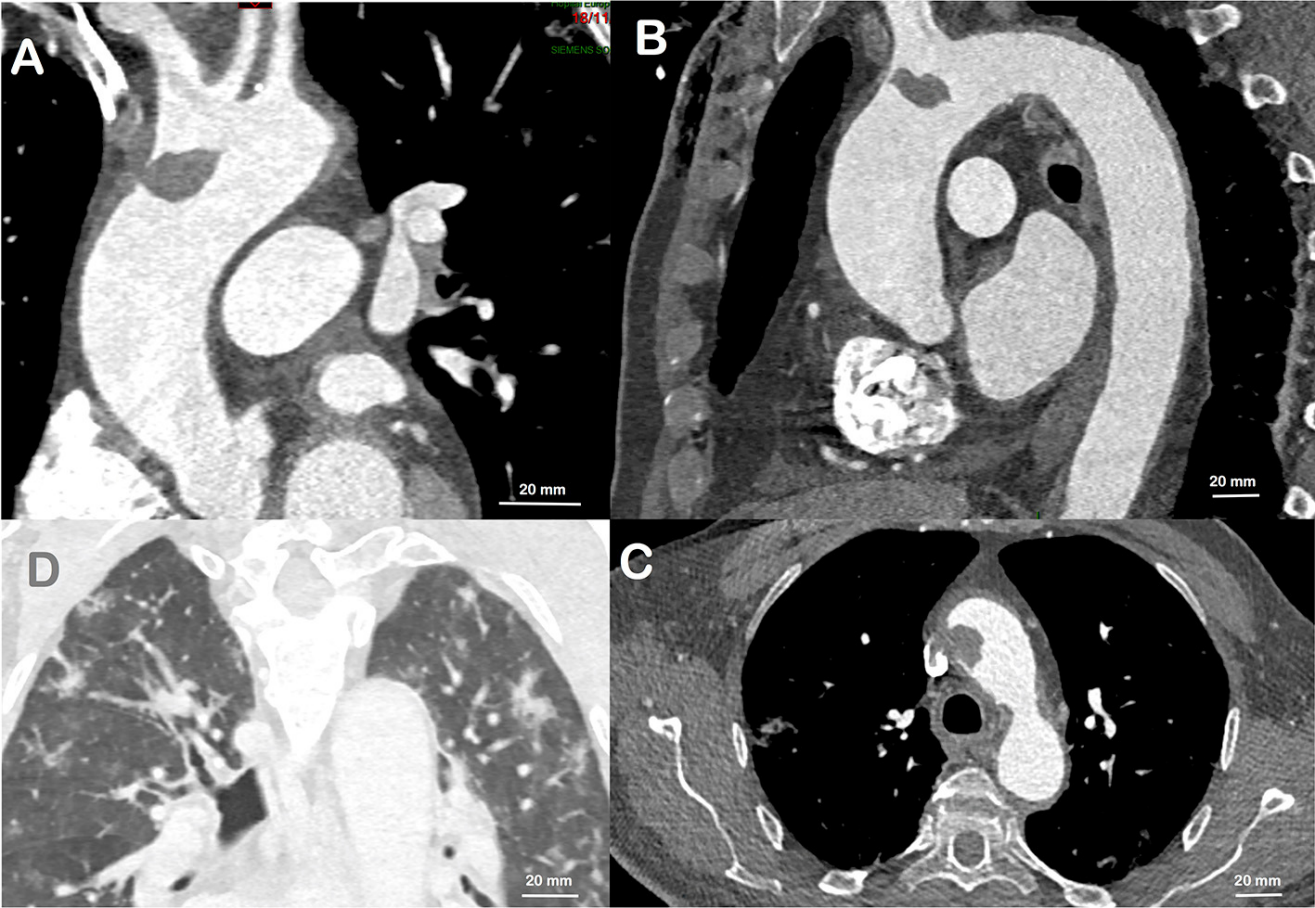
CT scan performed prior to cardiac surgery. A large thrombus at the base of the brachiocephalic trunk is visualized, and attached only to a short area on the aortic wall, in all views: sagittal **(A)**, coronal **(B)**, and transverse **(C)**. Bilateral Ground-glass opacities of the lungs in relation with the COVID-19 **(D)**.

**Figure 2:**
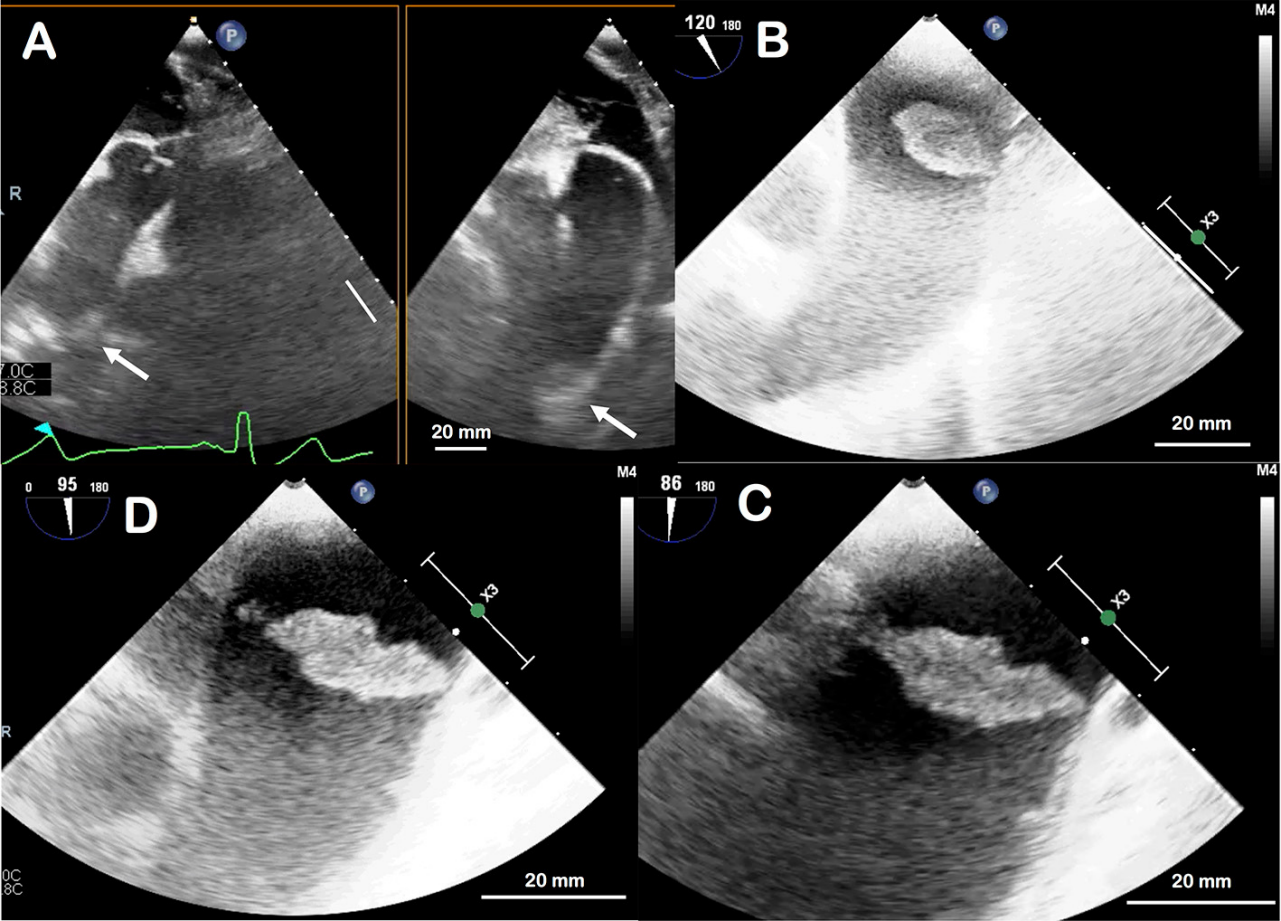
Transoesophageal ultrasound appearance of the thrombus in the aortic arch. Not visible on the initial part of the ascending aorta, it appears only in deep transgastric view by aligning the thoracic aorta **(A)**; white arrow indicates the thrombus. It is located 6 cm far from the aortic valve. The thrombus is then well-visualized by removing the ultrasound probe and aligning the aortic arch **(B)**. By zooming in on the thombus, it appears as a large, mobile and irregular thrombus, attached to a small base at the upper part of the aortic arch **(C,D)**.

**Figure 3:**
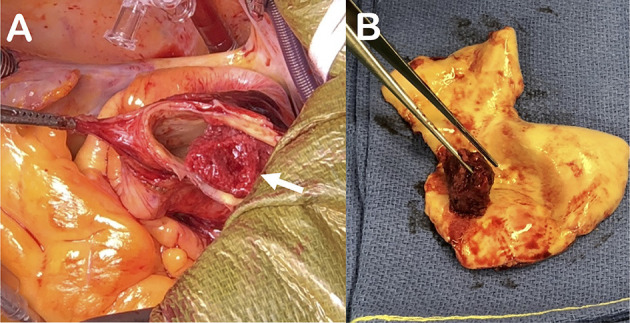
Peroperative aspect of the thrombus visualized attached to the aortic wall at the bottom of the brachiocephalic artery [(white arrow, **(A)**]. Resected aspect of the friable thrombus adherent to the aortic wall on a short base. The aortic inner wall is smooth without any plaque anfractuosity **(B)**.

**Figure 4:**
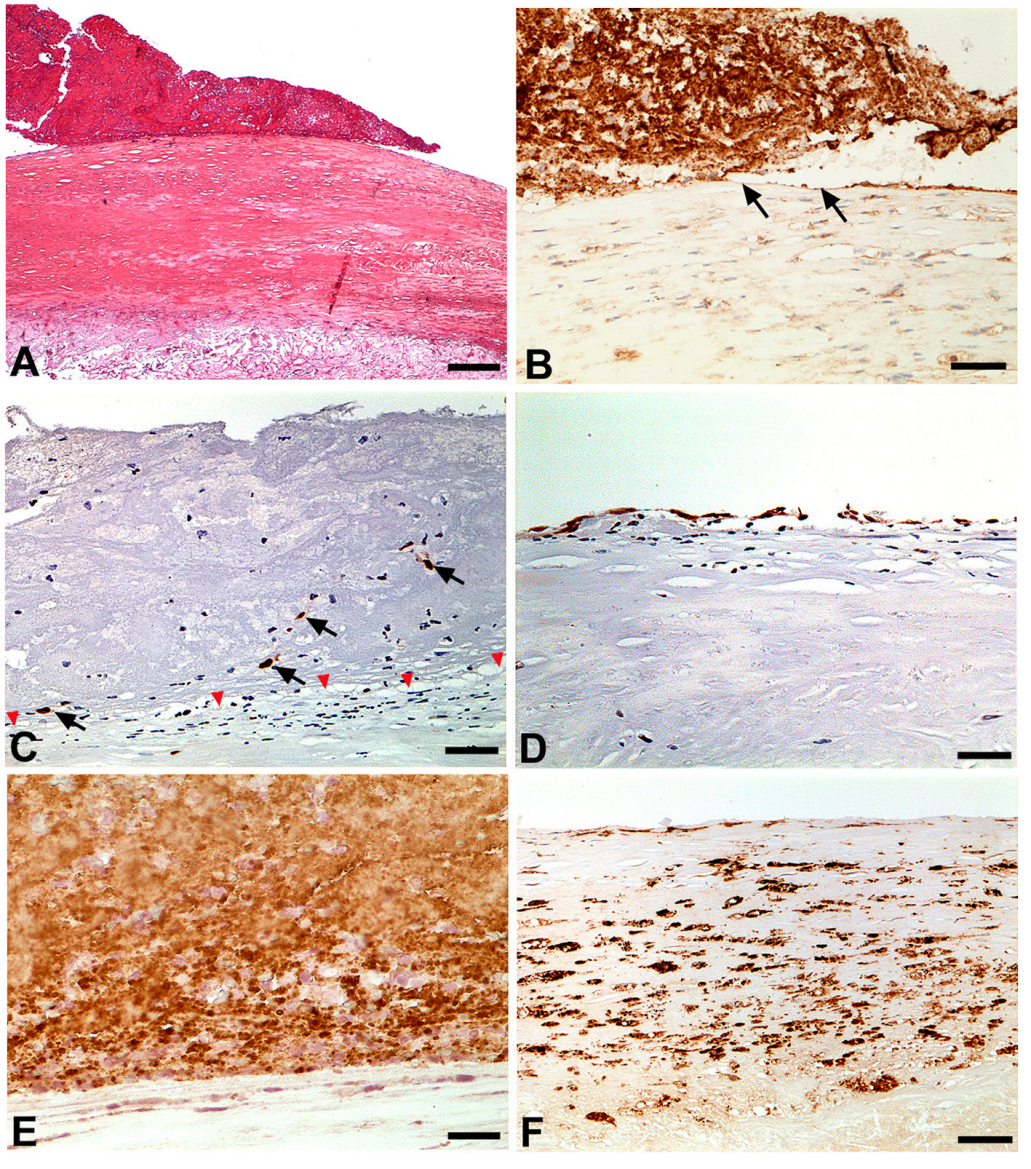
**(A)** The thrombus is lying on the thick fibrous cap of a fibrolipid plaque without any ulceration. This pattern is consistant with plaque erosion (H&E; Bar = 100 μm); **(B)** CD31 labeling shows the loss of endothelial cells below and adjacent to the thrombus (black arrows). The thrombus is also labeled given it includes large amount of platelets (CD31 immunohistochemistry; Bar = 10 μm); **(C)** The transcription factor ERG used as an endothelial cell marker shows the complete loss of endothelial cells below the thrombus leaving the intima devoid of endothelial cells (red arrowhead). Note that the thrombus embeds a few detached endothelial cells (arrows) (ERG immunohistochemistry; Bar = 10 μm); **(D)** ERG labeling exhibits a continuous endothelial cell layer in areas free of thrombus (ERG immunohistochemistry; Bar = 10 μm); **(E)** CD61 labeling shows that the thrombus is rich in platelets (CD61 immunohistochemistry; Bar = 5 μm); **(F)** Fibrous cap inflammation with macrophage infiltration is presented here in an area without thrombus (CD68 immunohistochemistry; Bar = 20 μm).

The patient reported unusual and fluctuating headaches 4 days earlier with a climax within the last 48 hours immediately followed by a sensory and motor loss in his left arm. He fully recovered from those neurological deficits and later went to the emergency department, off-delay for a potential thrombolysis. The clinical examination did not reveal any sensory or motor neurological deficit, the ECG was normal with sinus rhythm and the cerebral computed tomography (CT) scan was normal. MRI could not be performed due to presence of the carotid baroreceptor stimulation device. Although confirmation between a transient ischaemic attack and a stroke could not be proven, the diagnosis of stroke was retained due to the prolonged deficit and the normality of the early CT scan. The patient was immediately admitted and treated with aspirin. Biologically, sodium and potassium levels were normal, creatinine 112 μmol/L, C-reactive protein was elevated at 13.4 mg/L, hemoglobin 156 g/L, platelets 292 G/L, leucocytes 6.2 G/L. There was no obvious coagulation disorder, with a prothrombin time at 13.2 s (control at 13.2 s), an activated partial thromboplastin time at 29 s (control at 30 s), and a fibrinogen at 3.4 g/L. The investigations were completed at Day 2 with carotid and intracranial Duplex ultrasound, reporting the absence of significant stenosis of the carotid, vertebral or intracranial arteries. A CT-angiogram of the aorta revealed a large floating thrombus at the base of the brachiocephalic trunk (Figure 1), measuring 17.4 × 13.8 mm, confirming the embolic origin of stroke. In addition, bilateral multiple ground-glass opacities of the lungs were found and described as typical of pulmonary COVID-19. A SARS-CoV-2 infection was then confirmed by RT-PCR testing on a nasopharyngeal swab. Therapeutic anticoagulation with intravenous heparin was immediately started. After a multi-disciplinary discussion, the decision was made to perform an open aortic replacement surgery the same day because of the symptomatic thromboembolic event with recent cerebral infarction and the potential harmfulness of the thrombus due to its size. Although medical treatment alone has been reported to be effective in some cases (10, 11), surgical management was chosen here because of the risk of stroke recurrence and because such surgery is often offered with limited complications in aortic thrombus (12, 13). Peroperative trans-esophageal ultrasound showed good left ventricular function with normal mitral and aortic valves. There was neither ascending aorta dilatation nor aneurysm of the interauricular septum or patent foramen ovale after bubble study. A mobile thrombus was observed at the initial part of the aortic arch (Figure 2). It was attached to a small area of the upper aortic wall and had an irregular surface. Surgery was performed by median sternotomy. The cardiopulmonary bypass was established between the right atrium and the right axillary artery. Under selective antegrade cerebral perfusion and mild (28°C) systemic hypothermic circulatory arrest, the aorta was opened and the thrombus was found at the base of the brachiocephalic trunk (Figure 3). The entire ascending aorta was resected, removing the thrombus and its site of implantation, and replaced by a 30 mm diameter Dacron tube (Perouse Medical). The patient was extubated at Day 3 with oxygen support by Optiflow® (50% FiO2). Treatment with Dexamethasone 6 mg was introduced for a period of 10 days, associated to therapeutic level unfractionated heparin. The post-operative follow-up was simple and the patient fully recovered and returned home. The patient was treated with therapeutic anticoagulation because of the aortic thrombus but also because of the occurrence of post-operative atrial fibrillation, which was reduced by amiodarone. Unfractionated heparin was then replaced with warfarin. The assessment of haemostasis abnormalities revealed no Factor II 20210G/A mutation nor Factor V Q506 mutation, without deficiency of antithrombin, protein C, or protein S, and no lupus anticoagulant. Anticardiolipin and anti-β2 glycoprotein I antibodies assays were negative. At histology, the aorta showed several atherosclerotic plaques with plurifocal fibrous cap inflammation. There was no pronounced inflammation of the plaque, except for an isolated macrophage infiltration (Figure 4). At sites of thrombosis no plaque ulceration was present but endothelial cell desquamation was observed consistent with plaque erosion (Figure 4). The thrombus was platelet-rich and contained desquamated endothelial cells (Figure 4). Lastly, the SARS-CoV-2 RT-PCR testing performed on the thrombus and the aortic wall came back negative.

## Discussion

We report the case of a mobile thrombus of the initial part of the aortic arch, most likely responsible for a cerebral infarction associated and potentially subsequent to a slightly symptomatic SARS-CoV-2 infection. Pathology analysis showed the presence of a large thrombus attached to an extensive eroded atherosclerotic plaque. Cases of arterial thrombosis linked to COVID-19 seem to be favored by an underlying atherosclerosis, affecting patients at high cardiovascular risk, particularly those with high blood pressure (14). Arterial thrombus in COVID-19 may thus be linked to a destabilization of atherosclerotic plaques. However, the precise mechanism is currently debated. It is indeed suggested that the systemic inflammation triggered by the COVID-19 is associated with inflammation within atherosclerotic plaques (15, 16). Nevertheless, in our case, the atherosclerotic plaque appeared to be stable, with a thick fibrous cap, and without intra-plaque hemorrhage. The thrombus formation appeared more likely to relate to a very localized endothelial injury, explaining the appearance of the thrombus as “lying” on the plaque at the level of endothelial denudation areas. Although COVID-19 was reported to be a prothrombotic condition (1), the patient had no overt haemostasis disorder and the SARS-CoV-2 RT-PCR performed on the thrombus was negative. The mechanism of minimal superficial lesion of stable atherosclerotic plaques by endothelial injury is supported by the endotheliitis associated with COVID-19 already reported in the pulmonary arteries (17, 18). Moreover, in a rabbit model, overexpression of angiotensin conversion enzyme 2 (ACE2) resulted in stabilized atherosclerotic plaques. Authors suggested that the mechanism could probably due to the conversion of vasoconstrictive angiotensin II to vessel protective angiotensin 1–7 (19). As ACE2 is the molecular doorway to SARS-CoV-2 to enter cells it could explain that stable plaque may increase endothelial cells sensitivity to SARS-CoV-2. This possible mechanism should be even more alarming as it is likely to cause thrombosis at arterial segments were unstable atherosclerotic plaques are scarce, like in the initial part of the aortic arch. Nevertheless, cases of arterial thrombosis associated with COVID-19 remain infrequent. In the meta-analysis of Tan et al. (20), the overall incidence of arterial thromboembolic events among COVID-19 patients was 3.9%, much less than the 14.7% for venous thromboembolic events. SARS-CoV-2 infection alone appears to be the trigger for thrombosis, initiated on an atherosclerotic arterial wall, and not uniquely by creating a state of hypercoagulability. Therefore, patients with atherosclerotic plaques represent a population at risk for arterial thrombus. This should not be underestimated, even in the presence of asymptomatic COVID-19.

## Data Availability Statement

The original contributions presented in the study are included in the article/Supplementary Material, further inquiries can be directed to the corresponding author/s.

## Ethics Statement

Ethical approval was not provided for this study on human participants because Case report. The patients/participants provided their written informed consent to participate in this study.

## Author Contributions

GG, AD, and TM wrote the document. RE performed the histological analysis. MA, AB, and PA performed the aortic surgery. NG and DS performed the biological analysis. All authors interpreted the data, drafted and revised the manuscript, and approved the final version.

## Conflict of Interest

The authors declare that the research was conducted in the absence of any commercial or financial relationships that could be construed as a potential conflict of interest.

## Publisher's Note

All claims expressed in this article are solely those of the authors and do not necessarily represent those of their affiliated organizations, or those of the publisher, the editors and the reviewers. Any product that may be evaluated in this article, or claim that may be made by its manufacturer, is not guaranteed or endorsed by the publisher.

## Supplementary Material

Supplementary Data 1 Transoesophageal echocardiography movie.
